# Dataset yielded by an assessment of personality disorders among young French adults using the Inventory of Personality Organization

**DOI:** 10.1016/j.dib.2024.110806

**Published:** 2024-08-08

**Authors:** Franck Rexand-Galais, Lucas Pithon

**Affiliations:** a CLiPsy research unit, Angers University, SFR CONFLUENCES, Angers, France; b Spaces and Societies (ESO) research unit, Angers University, CNRS, SFR CONFLUENCES, Angers, France

**Keywords:** Inventory of Personality Organization, PDQ4+, DSM, Personality, Personality disorders, Personality disorder clusters, Young adults

## Abstract

Data on personality disorders in young French adults were collected using the validated French version of the Inventory of Personality Organization (IPO-fr). Respondents also completed the Personality Diagnostic Questionnaire-4th Edition (PDQ-4+). The sample comprised 607 students aged 18-26 years enrolled on a variety of courses at Angers University (France). Of these, 170 had a personality disorder according to the PDQ-4+, and their data were used to define IPO cut-off scores for Cluster A, B or C personality disorders. The data are stored in a comma-separated value format that can be easily downloaded from a Mendeley data repository (https://data.mendeley.com/datasets/tv6w6yyfy8/4). The data concerned by this article can be identified at this address under the name “Study 3”.

Specifications Table

**Table:** 

Subject	Psychology
Specific subject area	Clinical Psychology, Personality Assessment, Personality Disorders
Data format	Raw, Analyzed
Type of data	Tables
Data collection	Responses from 607 young French adults to the French version of the Inventory of Personality Organization (IPO-fr). Responses from the same 607 respondents to the French version of the Personality Diagnostic Questionnaire - 4th Edition (PDQ-4+): self-report questionnaire and Clinical Significance Scale. Basic demographic information (gender and age) was collected from all respondents. These data were collected in various departments of Angers University (Angers, France): 182 first- and second-year students from the Department of Psychology and 425 first- and second-year students from the Departments of History, Language Studies and Physical and Social Geography. Respondents receive no compensation for their voluntary participation. No exclusion criteria were applied: no exclusion was necessary, as the only exclusion criterion stipulated in the protocol approved by the Research Ethics Committee of Angers University (= age between 18 and 30) did not need to be applied to the sample. The sample also complies with ethical recommendations (Research Ethics Committee of Angers University: registration no UA-CER- 2021-09): the respondents had no subordinate relationships (e.g. teacher-to-teacher relationships) or other relationships providing potential sources of conflict of interest with the researchers. The age and gender ratios observed in this study were compared: they are typical of those observed in the French student population.
Data source location	Country: France.
Data accessibility	Repository name: Mendeley Direct URL to data: https://data.mendeley.com/datasets/tv6w6yyfy8/4 Accessibility information: The data presented in this article is directly identifiable under the name “Study 3” at the URL indicated above.
Related research article	L. Pithon, F. Rexand-Galais (2023) French version of the Inventory of Personality Organization (IPO-fr): Psychometric properties in young adults. Current Issues in Personality Psychology. https://doi.org/10.5114/cipp/174519

## 1 Value of the Data

• This dataset provides an effective and coherent alternative to the diagnosis of personality disorders based solely on DSM criteria, which have numerous limitations [1,2]. It is useful because it establishes thresholds that will facilitate the diagnosis of personality disorders in the French population, based on the use of the validated French version of the Personality Organization Inventory (IPO-fr) [3], which compensates for some of the DSM's limitations. These data are an effective tool for establishing a combined assessment.
• These data can be used in a dimensional protocol to help identify the presence or absence of a personality disorder. They distinguish between Clusters A, B and C on the basis of scores on the IPO-fr.
• These data also provide information on the prevalence of personality disorders in a population of young adults, as respondents also completed the French version of the Personality Diagnostic Questionnaire-4th Edition (PDQ-4+) [4].
• This dataset combining the scores of both clinical and nonclinical respondents on the IPO-fr and French PDQ-4+ can be used as part of a lifelong approach to personality. It can also be included in meta-analyses.

## 2 Data Description

This dataset was built with the purpose of determining valid cut-off scores for young French adults on the IPO-fr [1]. We collected responses to this self-report questionnaire [3] from 607 students aged 18-26 years. Items were rated on a 5-point Likert scale ranging from 1 (*Never true*) to 5 (*Always true*). These students also completed the PDQ-4+ to detect DSM-5 personality disorders, corroborated by its Clinical Significance Scale [4,5]. Results indicated that 170 of them had a personality disorder. The scores of these 170 students on the PDQ-4+ were used to establish cut-off scores on the IPO-fr for disorders in Clusters A (= paranoid, schizoid, and schizotypal), B (= antisocial, borderline, histrionic, and narcissistic), and C (= active-avoidant, obsessive-compulsive, and dependent).

• Table 1 shows the sample's demographic data and clinical characteristics according to PDQ-4+ scores.

**Table 1:** Demographic data for the total sample (*N* = 607) and clinical characteristics identified using the Personality Diagnostic Questionnaire-4th Edition (PDQ-4+).

Age in years (*SD*)	20.01 (1.98)
Female (%)	470 (80.7)
Personality disorders according to PDQ-4+ (%)	
No personality disorder	237 (39)
Any PD	370 (61)
Any Cluster A personality disorder Paranoid Schizoid Schizotypal	139 (22.9) 46 (7.6) 101 (16.6) 44 (7.2)
Any Cluster B personality disorder Histrionic Narcissistic Borderline Antisocial	194 (31.9) 28 (4.6) 37 (6.1) 120 (19.8) 10 (1.6)
Any Cluster C personality disorder Avoidant Dependent Obsessive-Compulsive	296 (48.8) 156 (25.7) 65 (10.7) 74 (12.2)
Depressive* Passive-Agressive*	148 (24.4) 28 (4.6)
Personality disorders after Clinical Significance Scale (%)	
No personality disorder Any personality disorder Cluster A personality disorder Cluster B personality disorder Cluster C personality disorder	437 (72) 170 (28) 13 (2.1) 69 (11.4) 89 (14.7)

⁎ Our classification method is based on the PDQ-4+ Scoring Form: depressive and passive-aggressive personalities are separated from A, B or C cluster disorders.
• Table 2 shows the IPO-fr scores of the 170 respondents with a personality disorder, according to their pathological profile (Cluster A, B or C).

**Table 2:** Descriptive statistics for Inventory of Personality Organization scales according to PDQ-4+ clusters.

	Cluster A (*n* = 13)		Cluster B (*n* = 68)		Cluster C (*n* = 89)	
Scale	Mean	*SD*	Mean	*SD*	Mean	*SD*
PD/ID	119.2	13.5	90.5	10.5	61.5	12.9
RT	33.5	4.5	19.13	5.8	13.9	2.4

*Note.* PD/ID = Primitive Defenses and Identity Diffusion; RT = Reality Testing.
• Table 3 compares the IPO-fr scores of the two most distinct groups (Clusters A and C) according to their receiver operating characteristic (ROC) curves and areas under the curve (AUCs).

**Table 3:** Areas under the curve (AUCs) for the Inventory of Personality Organization scales according to personality disorder cluster (A or C).

Variable	AUC	*SE*	95 % CI
Cluster A			
PD/ID	0.988	0.007	0.974, 1.000
RT	0.989	0.008	0.973, 1.000
Cluster C			
PD/ID	0.973	0.009	0.954, 0.991
RT	0.809	0.035	0.740, 0.877

*Note. N* = 170. CI = Confidence interval; PD/ID = Primitive Defenses and Identity Diffusion; RT = Reality Testing.
• Table 4 shows the IPO-fr cut-off scores for Cluster A and C personality disorders. Several factors were compared: sensitivity, specificity, positive and negative predictive values, positive and negative likelihood ratios, and Youden's index (fewer false positives and false negatives).

**Table 4:** Cut-off scores and accuracy indices for Clusters A and C based on Inventory of Personality Organization scales.

Scales	Criteria	Sensitivity	Specificity	Youden's index	+LR	-LR	+PV	-PV
Cluster A PD/ID RT Cluster C PD/ID RT	≥ 101 ≥ 25 ≤ 73 ≤ 18	1.00 1.00 0.831 0.989	0.930 0.892 0.978 0.932	0.930 0.892 0.809 0.921	14.273 9.235 33.674 2.356	0.000 0.000 0.173 0.019	0.542 0.433 0.974 0.721	1.000 1.000 0.840 0.979

*Note.* PD/ID = Primitive Defenses and Identity Diffusion; RT = Reality Testing; +/-LR = positive/negative Likelihood Ratios; +/-PV = positive/negative Predictive Values.
• Table 5 displays IPO-fr cut-off scores for all three clusters of personality disorders (A, B, and C).

**Table 5:** Cut-off scores for Clusters A, B and C based on Inventory of Personality Organization scales.

Scales	Cluster C	Cluster B	Cluster A
PD/ID RT	≤ 73 ≤ 18	≥ 74-100 ≤ ≥ 19-24 ≤	≥ 101 ≥ 25

*Note.* IPO = Inventory of Personality Organization; PD/ID = primitive defenses and identity diffusion; RT = reality testing.

## 3 Background

Scientific validation of the French version of the Kernberg Inventory of Personality Organization (IPO-fr) was carried out in related research [3]. This research has established the relevance of the IPO-fr as a reliable and brief instrument for assessing individual personality. It could make a major contribution to screening for personality pathologies in the French population. This dataset is based on a more precise inclusion of data than the related research and establishes the practical modalities of this assessment. It provides cut-off scores for the diagnosis of personality disorders, taking into account the different personality clusters (A, B and C) identified by the DSM-5.

## 4 Experimental Design, Materials and Methods

### 4.1 Design

This study was approved by the institutional review board of Angers University (registration no UA-CER-2021-09). In the presence of the clinical psychologists who developed this research, these data were anonymously collected between 26 November 2021 and 31 September 2022. After being given detailed written and oral background information, all participants provided their written informed consent in accordance with the French Code of Ethics for Psychologists [6]. The purpose of this study was to collate responses to the IPO-fr [3], responses to the PDQ-4+ and its Clinical Significance Scale [4,5], and basic demographic data (gender and age). These data were then cross-referenced, to produce IPO-fr cut-off scores, thereby enabling clinicians to distinguish between Cluster A, B and C personality disorders.

### 4.2 Material

The IPO-fr [3] is a 40-item self-report questionnaire. It measures the personality dimensions identified by Otto Kernberg [7,8]: Primitive Defenses (= mechanisms such as dissociation and denial vs more mature and elaborate defenses); Identity Diffusion (poorly integrated identity owing to inability to develop nuanced, complex and stable perceptions of self and others and preventing emotion regulation vs integrated identity); and Reality Testing (= capacity vs inability to maintain contact with reality).

The IPO-fr comprises two scales: PD/ID (30 items), and RT (10 items). Items are rated on a 5-point Likert scale ranging from 1 (*Never true*) to 5 (*Always true*). Scores on this questionnaire allow respondents’ personality to be placed on the normal-pathological continuum, isolating the more or less prevalent (i.e. pathological) presence of PD, ID, and RT. The level of personality organization is determined according to scores on the two IPO-fr scales [9]. The IPO-fr has good internal consistency in its two subscales: Cronbach's alpha ranges from acceptable (α > 0.70) for the RT scale to good (α > 0.87) for the PD/ID scale.

The 99-item PDQ-4+ is a self-report questionnaire based on the Personality Disorders section of the DSM-5. It assesses 10 specific Axis-II personality disorders: Cluster A includes paranoid (7 items), schizoid (7 items) and schizotypal (9 items) personalities; Cluster B groups antisocial (8 items), borderline (9 items), histrionic (8 items) and narcissistic (9 items) personalities; Cluster C contains avoidant (7 items), dependent (8 items) and obsessive-compulsive (8 items) personalities. The PDQ-4+ has been translated into French [4] and validated as a reliable tool for identifying personality disorders [5]. It is accompanied by a Clinical Significance Scale that enables clinicians to eliminate false positives. It is widely utilized as a screening instrument for personality disorders, particularly among nonclinical populations [10]. For Clusters A, B and C, the Cronbach's alpha of this study is good (α > 0.80).

### 4.3 Participants

The sample comprised 607 young adult respondents aged 18-26 years (Mage = 19.38, SD = 1.93). Respondents are first- and second-year students from the Department of Psychology (*n* = 182) and from the Departments of History, Language Studies and Physical and Social Geography (*n* = 425). Average age and gender representation are in accordance with data observed in student groups in Psychology as well as in Arts and Humanities in France [11]. Of the 607 respondents, 170 had a personality disorder as defined by the DSM-5: 138 women (*M*_age_ = 19.34 years, *SD* = 1.88, range = 18-26) and 32 men (*M*_age_ = 19.53 years, *SD* = 2.17, range = 18-25 years).

### 4.4 Methods, measures and data analysis

Respondents were diagnosed using the PDQ-4+ and its Clinical Significance Scale [4], and grouped according to the organization of personality disorders in the DSM-5.

The Clinical Significance Scale is an additional diagnostic tool for personality disorders. It enables clinicians to detect false positives. Taking the form of a short interview (approx. 20 min), this scale verifies that the respondent has not made a mistake in rating the items. In line with the DSM, it ensures that the disorders highlighted by the questionnaire are significant and stable, that is, the traits have been present for several years, are not due to an Axis-I disorder, and have a direct and real impact on the respondent's life (e.g., relationship with school, work, interpersonal relations).

This interview significantly reduced (−32.9 %) the proportion of respondents with a personality disorder according to the PDQ-4+, such that the final proportion was 28 %. These 170 participants were divided into Clusters A, B or C according to the nature of the personality disorders diagnosed using these two tools.

Table 2 shows the IPO-fr scores for each PDQ-4+ cluster.

Based on analysis of the ROC curves and AUCs (see Table 3), cut-off scores were defined for the personality disorder clusters (Table 4). As ROC curves assess the performance of a binary classifier, they display the contrast between the two most distinct groups (here, Clusters A and C) (Table 5).

To define the cut-off scores for each cluster, we compared the following factors across both scales: sensitivity, specificity, positive and negative predictive values, positive and negative likelihood ratios, and Youdenʼs index (Table 4). For each of these indices, the higher the value the more precise the distinction, and therefore the more effective the test. ROC curve analysis (i.e. assessment of performance of a binary classifier) produced cut-off scores for Clusters A and C.

The accuracy of the classification indices (sensitivity, specificity, positive and negative predictive values, positive and negative likelihood ratios, and Youden's index) revealed that (Table 4):

• Scores below or equal to 73 for PD/ID and 18 for RT were representative of Cluster C.
• Scores above or equal to 101 for PD/ID and 25 for RT were representative of Cluster A.

The binary classification based on the ROC curves and AUCs (Table 3) produced cut-off scores for Clusters A and C (Table 3), the accuracy indices having been assessed for these two clusters. Consequently, the cut-off scores for Cluster B came in between the cut-off scores for the other two clusters (Table 5):

• Scores between 73 and 101 for PD/ID, and between 18 and 25 for RT were representative of a Cluster B personality disorder.

## Limitations

The main shortcoming of this dataset is the low proportion of Cluster A respondents in our sample (*n* = 13). These data therefore need to be consolidated by future studies. While the relevant cut-off scores should not be called into question, they should be treated with caution for Cluster A respondents.

## Ethics Statement

This study was approved by the institutional review board of Angers University (registration no UA-CER-2021-09). Respondents provided their written informed consent, in accordance with the French Code of Ethics for Psychologists [6].

## CRedit Author statement

**Franck Rexand-Galais**: Conceptualization, Data curation, Formal analysis, Methodology, Project administration, Resources, Supervision, Validation, Writing – review & editing. **Lucas Pithon**: Conceptualization, Data curation, Investigation, Formal analysis, Methodology, Software, Visualization, Writing – original draft.

## Data Availability

Dataset assessing three samples of French university students to 5 personality tests: IPO, PDQ-4+, PANAS, AQ and HADS (Original data) (Mendeley Data)
